# Fatty Acid Biosynthesis Inhibition Increases Reduction Potential in Neuronal Cells under Hypoxia

**DOI:** 10.3389/fnins.2016.00546

**Published:** 2016-11-30

**Authors:** Stephen A. Brose, Svetlana A. Golovko, Mikhail Y. Golovko

**Affiliations:** Department of Biomedical Sciences, University of North DakotaGrand Forks, ND, USA

**Keywords:** hypoxia, reduction and oxidation potential, lactic acid, NAD, NADP, fatty acids, lipid biosynthesis

## Abstract

Recently, we have reported a novel neuronal specific pathway for adaptation to hypoxia through increased fatty acid (FA) biosynthesis followed by esterification into lipids. However, the biological role of this pathway under hypoxia remains to be elucidated. In the presented study, we have tested our hypothesis that activation of FA synthesis maintains reduction potential and reduces lactoacidosis in neuronal cells under hypoxia. To address this hypothesis, we measured the effect of FA synthesis inhibition on ${\text{NADH}}_{2}^{+}$/NAD^+^ and ${\text{NADPH}}_{2}^{+}$/NADP^+^ ratios, and lactic acid levels in neuronal SH-SY5Y cells exposed to normoxic and hypoxic conditions. FA synthesis inhibitors, TOFA (inhibits Acetyl-CoA carboxylase) and cerulenin (inhibits FA synthase), increased ${\text{NADH}}_{2}^{+}$/NAD^+^ and ${\text{NADPH}}_{2}^{+}$/NADP^+^ ratios under hypoxia. Further, FA synthesis inhibition increased lactic acid under both normoxic and hypoxic conditions, and caused cytotoxicity under hypoxia but not normoxia. These results indicate that FA may serve as hydrogen acceptors under hypoxia, thus supporting oxidation reactions including anaerobic glycolysis. These findings may help to identify a radically different approach to attenuate hypoxia related pathophysiology in the nervous system including stroke.

## Introduction

Despite the significant role for brain hypoxia in the development of many of pathophysiological conditions including stroke, traumatic brain injury, tumorigenesis, aging, and neurodegenerative diseases (Gallagher and Hackett, 2004; Wilson et al., 2009; Raymond et al., 2011; Clambey et al., 2012; Kirby et al., 2012; Lin et al., 2013), biochemical mechanisms for adaptation to hypoxia are still poorly understood. Recently, we have discovered a previously unknown and neuron-specific mechanism for utilizing anaerobic metabolism during hypoxia. We have found that hypoxic-stressed neurons have a unique response of dramatically increased fatty acid (FA) synthesis from glutamine and glutamate (Gln/Glu) (Brose et al., 2014). However, the biochemical significance of this pathway in neuronal adaptation to hypoxia is unknown. Previously, we have hypothesized a few mechanisms to address the importance for activated FA synthesis from Gln/Glu under hypoxia (Brose et al., 2014) including balancing Glu levels, protection against oxidative stress, and maintaining reduction potential with support of anaerobic glycolysis. In the present study, we have tested one of these hypotheses that FA SYNTHESIS supports anaerobic metabolism under hypoxia through accepting hydrogen from reduced cofactors (${\text{NADH}}_{2}^{+}$, ${\text{NADPH}}_{2}^{+}$, FADH_2_), thus maintaining reduction potential.

Under hypoxia, hydrogen accumulates on reduced cofactors (${\text{NADH}}_{2}^{+}$, ${\text{NADPH}}_{2}^{+}$, FADH_2_; Garofalo et al., 1988; Obi-Tabot et al., 1993; Foster et al., 2005), due to the decrease in O_2_ as its final acceptor. This results in a decreased ATP production through oxidative phosphorylation, and an increased ratio of reduced/oxidized cofactors in the cell and therefore, an increased reduction potential. The altered reduction potential has several devastating effects on cells including lactate accumulation as an alternative H_2_ acceptor and subsequent pH drop (Payen et al., 1996; Malisza et al., 1999; Zhang et al., 2006), increased formation of reactive oxygen species which damage lipids, proteins, and DNA (Magalhães et al., 2005), DNA modification through modulation of sirtuin Sirt1 activity (Lin et al., 2004), decreased rates of oxidation reactions such as glycolysis or Glu/Gln oxidation (McKenna, 2007), and a further decrease in ATP production (Pettit et al., 1975). Because FA synthesis consumes two hydrogens from reduced cofactors for each 2 carbons incorporated, we hypothesize that FA synthesis may have a role as a hydrogen acceptor from reduced cofactors under hypoxia, thus maintaining cellular reduction potential.

To address this hypothesis, we applied a previously validated *in-vitro* model for neuronal hypoxia using SH-SY5Y cells exposed to 19% (normoxia) or 1% (hypoxia) oxygen levels (Brose et al., 2014). FA synthesis was inhibited at two different steps in the biosynthetic pathway using tetradecyloxy-2-furoic acid (TOFA, inhibits Acetyl-CoA carboxylase Loftus et al., 2000) and cerulenin (inhibits FA synthase, Heiligtag et al., 2002; Lupu and Menendez, 2006). FA synthesis inhibition significantly increased ${\text{NADH}}_{2}^{+}$/NAD^+^ and ${\text{NADPH}}_{2}^{+}$/NADP^+^ ratios under hypoxia and resulted in increased lactic acid under both normoxic and hypoxic conditions. Importantly, FA synthesis inhibition caused cytotoxicity under hypoxia but not normoxia. These results indicate that FA may serve as hydrogen acceptors under hypoxia, thus supporting oxidation reaction including anaerobic glycolysis. These findings may help to identify a radically different approach to attenuate hypoxia related pathophysiology in the nervous system including stroke.

## Materials and methods

### Materials

SH-SY5Y cells were a gift from Dr. Colin Combs. All culture media and horse serum were purchased from Life Technologies (Grand Island, NY, USA). Fetal bovine serum (FBS) was purchased from Serum Source International (Charlotte, NC, USA). L-[U ^14^C] glutamaic acid (260 mCi/mmol) was purchased from PerkinElmer (Waltham, MA, USA). TOFA and cerulenin were purchased from Cayman Chemical (Ann Arbor, MI, USA). All other chemicals and solvents used were purchased from Fisher Scientific (Waltham, MA USA) and were LC-MS grade.

### Cell culture and hypoxic treatment

Cells were plated 3 days before the experiment on a six-well plate (Cellstar, Griner Bio-One, Monroe, NC, USA) at a density of 1.5 million cells per well. The cells were grown in Dulbecco's modified Eagle medium with nutrient mixture F-12 (DMEM/F-12) with 10% FBS and 5% horse serum at 37°C and 5% CO_2_.

The hypoxic treatment was as described earlier (Brose et al., 2014). Briefly, the cells were preconditioned by replacing the growth medium with serum-free minimum essential media (MEM) and incubating in 19% O_2_ (Normoxia) or 1% O_2_ (Hypoxia) in 5% CO_2_ at 37°C using nitrogen gas to purge the oxygen. After 24 h, the media was replaced with 2 mL of fresh serum-free MEM containing radiolabeled tracer (2 μCi [U-^14^C Glu]) and/or fatty acid synthesis inhibitor (TOFA, 2 μg/mL; cerulenin, 1 μg/mL) and returned to their respective incubation conditions for another 18 h. A short re-oxygenation during media change did not significantly affect the FA synthesis rate as was estimated using de-oxygenated media (data not shown).

### Lipid extraction and saponification

To measure incorporation of radiotracer into fatty acids, cellular lipids were extracted and saponified as described earlier (Brose et al., 2014). Briefly, the media was removed and the cells were washed twice with ice-cold phosphate buffered saline. After removing the final wash, 0.5 mL of methanol was added to the cells; they were scraped and transferred into a silanized with Sigmacote (Sigma Chemical Co., St. Louis, MO) screw top glass tube. Another, 0.5 mL of methanol was added, the plates were scraped again, and the solution was combined with the methanol solution. A Folch extract (Folch et al., 1957) was performed by adding an additional 1 mL of methanol and 4 mL chloroform. The mixture was soniciated using a probe sonicator (Model 150 Sonic Dismembrator, Fisher Scientific) and centrifuged at 2000 × g for 10 min. The supernatant was transferred into a new silanized screw top glass tube and was washed with 1.2 mL saline (0.9% sodium chloride). The extract was washed an additional two times with 1.2 mL chloroform:methanol:water (3:48:47). The extract was dried under nitrogen, re-dissolved in the saponification solution (180 μL methanol and 20 μl 5M potassium hydroxide in water) and heated to 60°C for 60 min to saponify. The samples were then neutralized with 20 μL 5 M hydrochloric acid in water. After neutralization, 780 μL of saline was added and the fatty acids were extracted with 2 mL hexane three times. The combined hexane extracts were evaporated and the fatty acids were re-dissolved in 1 mL of hexane. Radioactivity of an aliquot of the samples was measured in 10 mL Cytoscint (MP Biomedicals; Solon, OH, USA) using a scintillation counter (LS-6500, Beckman Coulter, Pasadena, CA, USA).

### Cytotoxicity

Cytotoxicity was measured as a percent of lactate dehydrogenase (LDH) released from the cells into the media using an enzymatic kit (BioVision, Milpitas, CA, USA). Media was collected, and the cells were lysed in 1 mL of the included lysis buffer. For both media and cell lysate, 10 μL was used for LDH measurement. Absorbance was measured at 450 nm using a Flexstation III plate reader (Molecular Devices; Sunnyvale, CA, USA). LDH released was calculated as (LDH_medium_/LDH_medium+cells_) × 100%.

### Lactic acid

Lactic acid was measured using 20 μL of media with a fluorescence-based enzymatic kit from Cayman Chemical. Fluorescence was measured with a Flexstation III plate reader using an excitation wavelength of 535 nm and an emission wavelength of 590 nm with a cutoff filter of 570 nm.

### NAD^+^/NADH_2_ and NADP^+^/NADPH_2_ measurement

Nucleotides were extracted from cells under ice-cold conditions by adding ice-cold 0.5 mL methanol:water (80:20) containing 0.1 mg/mL ethylenediaminetetraacetic acid and scraping the cells. The solution containing cells was transferred to a 1.5 mL microcentrifuge tube. Another 0.5 mL of the methanol:water solution was added to each well, the wells were scraped again, and the wash was combined with the previous wash. The combined solutions were sonicated, centrifuged at 10000 × g for 10 min at 4°C. The supernatant was transferred into a 2 mL microcentrifuge tube and was washed from lipids with 1 mL hexane 2 times. The remaining solution was evaporated in a vacuum concentrator. The residue was redissolved in 20 μL water and transferred into a silanized microvial insert (Microsolv, Eatontown, NJ USA part number 9502S-02ND) and 10 μL was injected into the LC-MS.

Nucleotides were separated on the same day using a HYPERCARB column (3 μm, 250 Å, 150 × 2.1 mm; Thermo Fisher Scientific; Waltham, MA, USA) maintained at room temperature. The LC system was a Waters AQUITY UPLC pump and a well plate autosampler (Waters; Milford, MA, USA). The autosampler temperature was 8°C. Solvent A consisted of water containing 2 mM ammonium acetate at pH 10 and solvent B consisted of acetonitrile containing 2 mM ammonium acetate at pH 10. The flow rate was 0.3 mL/min and the initial conditions were 97% A and 3% B. The initial conditions were held for 0.5 min. B was increased to 10% over 8 min and held for 2 min. B was then further increased to 25% over 2 min and held for 4 min. Finally, B was increased to 98% over 4 min and held for 7.5 min. B was then returned to the initial conditions over 0.5 min and held for 2 min.

Quantification of nucleotides was performed on a Waters Synapt G2-S quadrupole time-of-flight mass spectrometer (Q-TOF). The electrospray ionization was in negative ion mode as previously described (Brose et al., 2013). The cone voltage was 20 V with a capillary voltage of 1.51 kV. The source temperature was 110°C. The desolvation temperature was 350°C. The cone gas flow was 10 L/h, the desolvation gas flow was 1000 L/h and the nebulizer gas was 6bar. The analyzer was operated in the centroid sensitivity mode with an extended dynamic range with a resolution of 10,000. Mass correction was performed using leucine enkephalin (400 pg/μL, ACN: water, 50: 50) which was infused at 10 μL/min. The acquisition rate was 10 hertz. NAD^+^, ${\text{NADH}}_{2}^{+}$, NADP^+^, and ${\text{NADPH}}_{2}^{+}$ were quantified using m/z 662.1013, 664.1161, 742.0670, and 744.0833 Da, respectively. Instrument control, acquisition, and sample analysis was performed using MassLynx V4.1 software (Waters).

### Statistics

Statistical comparisons were determined using an ANOVA with Tukey's *post-hoc* test. Statistical significance was defined as < 0.05. Values are expressed as mean ± SD. GraphPad Prism 6 (GraphPad; San Diego, CA) software was used for statistical analysis.

## Results

**Figure 1:**
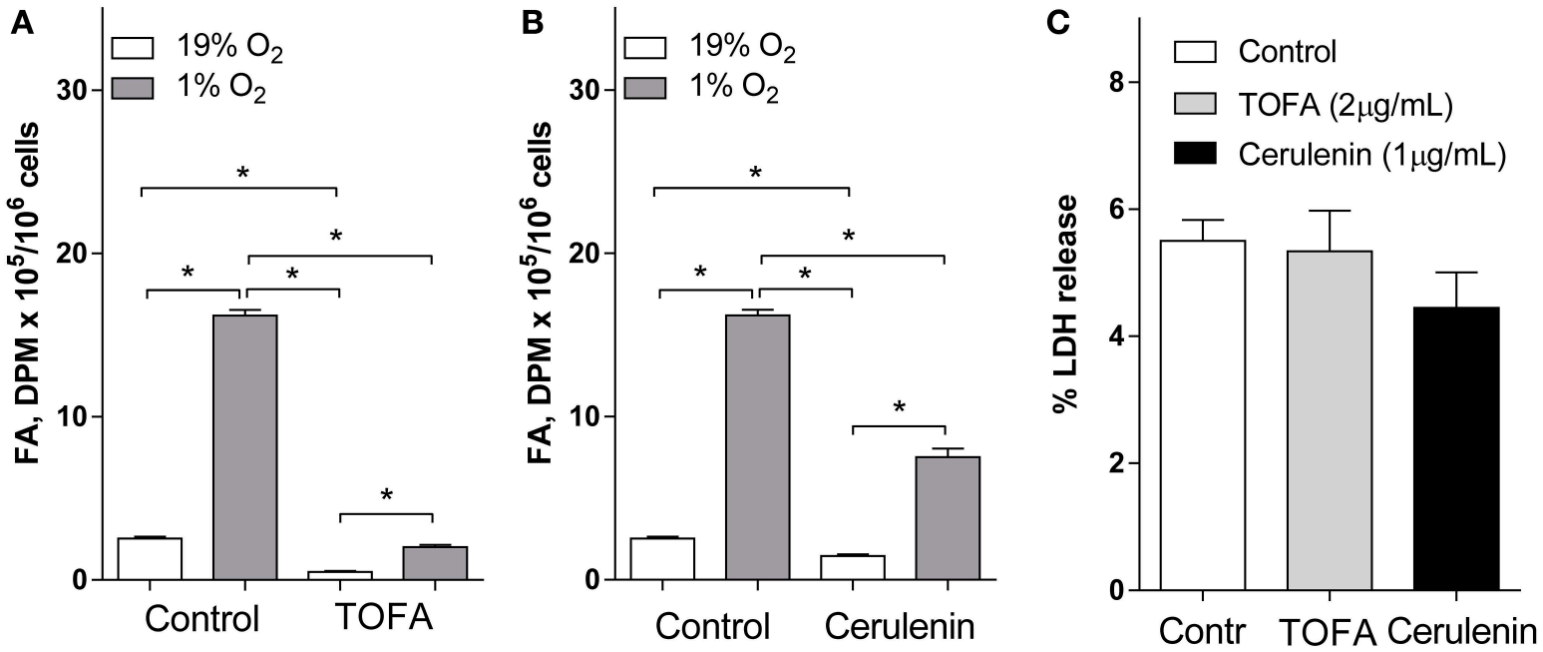
**Fatty acid synthesis from glutamate in SH-SY5Y cells was inhibited by TOFA and cerulenin under normoxia (19% O_2_) and hypoxia (1% O_2_) and did not cause toxicity under normoxia. (A,B):** SH-SY5Y cells were preconditioned in serum-free MEM for 24 h under normoxia or hypoxia. The media was replaced with a fresh media and the cells were pretreated with vehicle (control, 1 μL/mL DMSO), **(A)** TOFA (2 μg/mL), or **(B)** cerulenin (1 μg/mL) for 30 min. [U-^14^C] glutamate (2 μCi) was then added to the wells. The cells were incubated for another 18 h under normoxia or hypoxia. Fatty acid (FA) radioactivity was determined as described in the Materials and Methods. **(C)**: Percent of LDH released into the media was measured under normoxic (19% O_2_) conditions to confirm the inhibitors TOFA (2 μg/mL) and cerulenin (1 μg/mL) were not toxic at the concentrations used. ^*^-significantly different, *p* < 0.05. Values are mean ± SD, *n* = 3.

To address the role for increased FA synthesis under hypoxia, we use our previously validated model for neuronal cell hypoxia (Brose et al., 2014). Consistent with our previous results, FA synthesis from Glu was dramatically 6.4-fold increased in SH-SY5Y cells under 1% O_2_ (Figure 1). Next, we inhibited FA synthesis at the Acetyl-CoA carboxylase (TOFA, Loftus et al., 2000) or FA synthase (cerulenin, Heiligtag et al., 2002; Lupu and Menendez, 2006) reactions. The inhibitors TOFA and cerulenin were used at concentrations 5- and 3-fold above their IC_50_ values, respectively (Zhu et al., 2004; Wu et al., 2011), and significantly inhibited FA synthesis from Glu under both normoxic and hypoxic conditions (Figures 1A,B) while they were not toxic under normoxia (Figure 1C). Importantly, at the concentrations used, TOFA demonstrated a higher potency to inhibit FA synthesis under both normoxia (5.1-fold FA synthesis inhibition by TOFA compared to 1.7-fold inhibition by cerulenin) and hypoxia (8.1-fold FA synthesis inhibition by TOFA compared to 2.2-fold inhibition by cerulenin) (Figures 1A,B).

**Figure 2:**
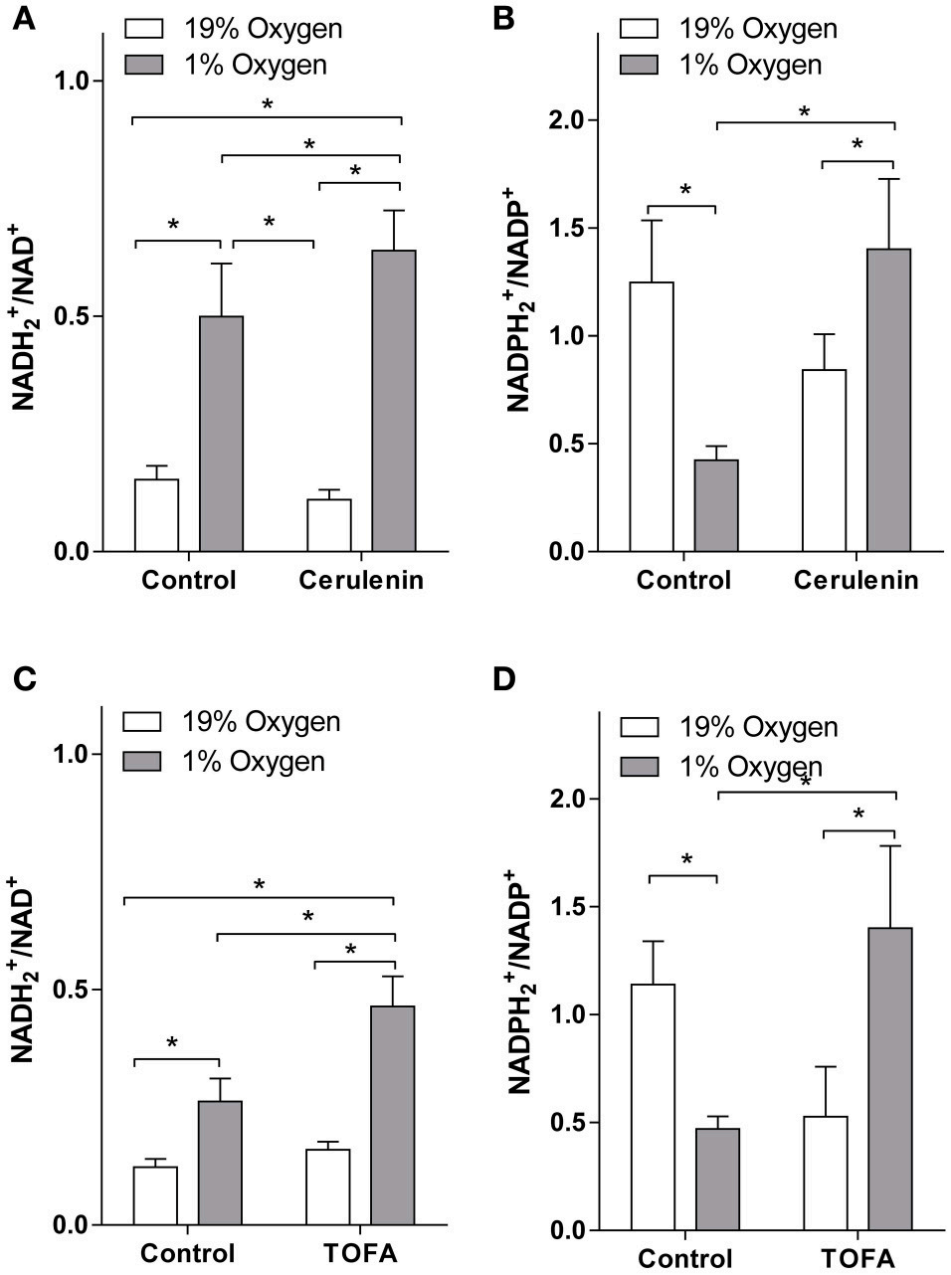
**${\text{NADH}}_{2}^{+}$/NAD^+^ and ${\text{NADPH}}_{2}^{+}$ /NADP^+^ levels under hypoxia are increased with fatty acid synthesis inhibition**. Inhibition of fatty acid synthesis in hypoxic SH-SY5Y cells increases the amounts of ${\text{NADH}}_{2}^{+}$ **(A,C)** and ${\text{NADPH}}_{2}^{+}$ **(B,D)** relative to their oxidized forms. SH-SY5Y cells were preconditioned in serum-free MEM for 24 h under normoxia or hypoxia. The media was switched to a fresh media and the cells were treated with vehicle (control, 1 μL/mL DMSO), cerulenin (1 μg/mL) **(A,B)**, or TOFA (2 μg/mL) **(C,D)** and were incubated for another 18 h under normoxia or hypoxia. Nucleotides were extracted with 80:20 methanol: water containing EDTA and were analyzed with LC-MS as described in the Materials and Methods. ^*^-significantly different, *p* < 0.05. Values are mean ± SD, *n* = 3.

To assay the effect of FA synthesis inhibition on cellular reduction potential, we applied a high resolution accurate mass LC-MS approach to measure ${\text{NADH}}_{2}^{+}$/NAD^+^ and ${\text{NADPH}}_{2}^{+}$/NADP^+^ ratios under normoxia and hypoxia (Figure 2). In the control (vehicle treated) cells, hypoxia resulted in an increased ${\text{NADH}}_{2}^{+}$/NAD^+^ ratio in both TOFA and cerulenin experiments. This is consistent with hypoxic conditions when O_2_ levels are insufficient to accept H_2_ from reduced cofactors through the electron transport chain. Slight differences in the ${\text{NADH}}_{2}^{+}$/NAD^+^ and ${\text{NADPH}}_{2}^{+}$/NADP^+^ ratios between experiments may be attributed to the differences between culture age and density because TOFA and cerulenin experiments were performed at different times, and the ${\text{NADH}}_{2}^{+}$/NAD^+^ ratio is closely linked to physiological and pathological states (Schwartz et al., 1974; Atzori et al., 1990; Zhang et al., 2006; Sun et al., 2012). Surprisingly, hypoxia decreased ${\text{NADPH}}_{2}^{+}$/NADP^+^ ratio. This is consistent with previous reports (Tribble and Jones, 1990; Gupte and Wolin, 2006; Kathagen-Buhmann et al., 2016) and may be associated with the depression of pentose-phosphate pathway (Gupte and Wolin, 2006; Kathagen-Buhmann et al., 2016). Alternatively, decreased ${\text{NADPH}}_{2}^{+}$ under hypoxia may be explained through significant increased FA synthesis that utilizes ${\text{NADPH}}_{2}^{+}$ as a cofactor.

Consistent with our hypothesis, both inhibitors significantly increased both ${\text{NADH}}_{2}^{+}$/NAD^+^ and ${\text{NADPH}}_{2}^{+}$/NADP^+^ ratios under hypoxia as compared to vehicle treated hypoxic cells (Figure 2). Similar to the effect on FA synthesis, TOFA had a stronger effect on ${\text{NADH}}_{2}^{+}$/NAD^+^ ratios (1.8-fold increase as compared to vehicle treated hypoxic cells) as compared to cerulenin (1.3-fold increase), while the effect on ${\text{NADPH}}_{2}^{+}$/NADP^+^ ratios was similar for both inhibitors (~3-fold increase as compared to vehicle treated hypoxic cells).

**Figure 3:**
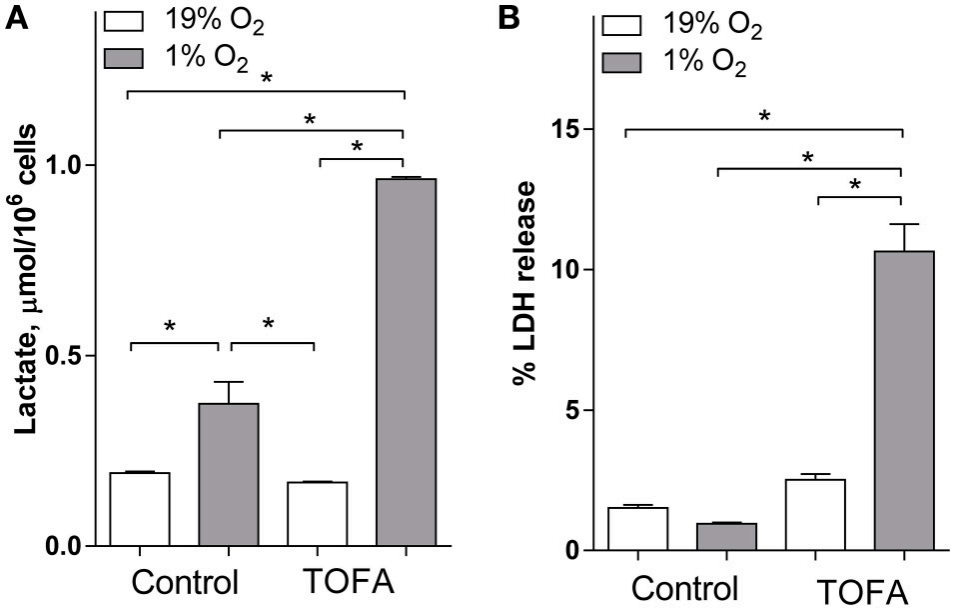
**Inhibition of fatty acid synthesis with TOFA in SH-SY5Y cells increases lactic acid levels and toxicity under hypoxia**. SH-SY5Y cells were preconditioned in serum-free MEM for 24 h under normoxia or hypoxia. The media was switched to a fresh media and the cells were treated with vehicle (control, 1 μL/mL DMSO) or TOFA (2 μg/mL). The cells were incubated for another 18 h under normoxia or hypoxia. **(A)**: Lactic acid was measured using an enzymatic fluorescent kit. **(B)**: Toxicity was determined through percent of LDH release which was measured using a colorimetric enzymatic kit. ^*^significantly different, *p* < 0.05. Values are mean ± SD, *n* = 3.

Because the ${\text{NADH}}_{2}^{+}$/NAD^+^ ratio is closely related to anaerobic glycolysis and lactic acidosis which are both activated under 1% hypoxia (Zhang et al., 2006), we assayed the effect of FA synthesis inhibition with TOFA on media lactic acid (Figure 3A). Consistent with anaerobic glycolysis activation under hypoxia, 1% O_2_ increased media lactic acid 1.9-fold. TOFA did not have an effect on lactic acid under normoxia, but significantly increased lactate under hypoxia 2.6-fold as compared to vehicle treated hypoxic cells (Figure 3A). Consistent with increased ${\text{NADH}}_{2}^{+}$/NAD^+^ and ${\text{NADPH}}_{2}^{+}$/NADP^+^ ratios, and increased lactoacidosis under hypoxia, TOFA dramatically increased cytotoxicity under hypoxia but not normoxia as measured by cellular LDH release (Figure 3B).

## Discussion

Despite the significant contribution of brain hypoxia in the development of many of pathophysiological conditions, biochemical mechanisms for neuronal adaptation to hypoxia are still not completely understood. Previously, using both primary neurons and neuronal cell lines, we have reported a novel response of neuronal cells to hypoxia through a dramatic increase in FA synthesis from Gln/Glu (Brose et al., 2014). However, the biological importance for this pathway has not been addressed.

To explain the role for increased FA synthesis under neuronal hypoxia, we have previously hypothesized few mechanisms that may have a complimentary protective role, including balancing Glu levels, protection against oxidative stress, and maintaining reduction potential with support of anaerobic glycolysis (Brose et al., 2014). In the current study, we have addressed the role for FA synthesis in supporting reduction potential as a potent acceptor of hydrogen under hypoxia.

The mechanism for alterations in reduction potential under hypoxia is well understood. Under hypoxia, hydrogen transfer from substrates to oxygen in the electron transport chain in mitochondria is decreased. This leads to accumulation of hydrogen on intermediate cofactors (${\text{NADH}}_{2}^{+}$, ${\text{NADPH}}_{2}^{+}$, FADH_2_; Garofalo et al., 1988; Obi-Tabot et al., 1993; Foster et al., 2005). Because the total pool of reduced and oxidized co-factors is unchanged, the level of oxidized cofactors is decreased, thus the ratio between reduced and oxidized cofactors is increased. As a result, ATP levels are decreased, while the reduced cofactors (${\text{NADH}}_{2}^{+}$, ${\text{NADPH}}_{2}^{+}$, FADH_2_) are significantly increased, limiting energy production and increasing reduced/oxidized cofactor ratio in cells. In addition, cells are unable to completely oxidize pyruvate and acetyl-CoA produced in glycolysis and glutaminolysis (McKenna, 2007). In line with this mechanism, and consistent with previous studies when 1% O_2_ was used to model hypoxia (Zhang et al., 2006), we observed a significant increase in the ${\text{NADH}}_{2}^{+}$/NAD^+^ ratio and lactic acid accumulation in control hypoxic cells (Figures 2, 3). However, similar to previous reports (Tribble and Jones, 1990; Gupte and Wolin, 2006; Kathagen-Buhmann et al., 2016), hypoxia decreased the ${\text{NADPH}}_{2}^{+}$/NADP^+^ ratio (Figure 2) which may be explained through the depression of the pentose-phosphate pathway(Gupte and Wolin, 2006; Kathagen-Buhmann et al., 2016). Alternatively, decreased ${\text{NADPH}}_{2}^{+}$ under hypoxia may be explained through significantly increased FA synthesis under hypoxia since FA synthesis utilizes ${\text{NADPH}}_{2}^{+}$ as a cofactor. Astrocytes and, to a lesser extent, neurons adapt to hypoxic conditions through switching to anaerobic glycolysis. However, under hypoxia anaerobic glycolysis leads to further accumulation of both reduced cofactors and lactic acid (Figure 3). This is consistent with attenuation of ${\text{NADH}}_{2}^{+}$ accumulation under hypoxia when glucose levels are decreased (Garofalo et al., 1988).

The altered reduction potential has several devastating effects on cells. It results in: **1**. Further lactate accumulation (as an alternative acceptor of hydrogen), and pH drop (Payen et al., 1996; Malisza et al., 1999; Zhang et al., 2006). Importantly, even under normoxia, astrocytic lactate production is very high to provide this key metabolite to neurons for energy metabolism (Hu and Wilson, 1997; Galeffi et al., 2007). Under hypoxia, neuronal oxidative potential is limited, decreasing lactate utilization; **2**. Reactive oxygen species formation with consequential damage to lipids, proteins, and DNA (Magalhães et al., 2005). This paradoxical phenomenon of hypoxia-induced oxidative stress is explained through increased mitochondrial reductive stress (Turrens et al., 1985; Duranteau et al., 1998). One of the additional mechanisms for increased oxidative damage under accumulation of ${\text{NADH}}_{2}^{+}$ is the formation of H_2_O_2_ that promotes reactive oxygen species formation (Circu and Aw, 2010). Intriguingly, the ${\text{NADPH}}_{2}^{+}$ to NADP^+^ ratio is decreased under hypoxia as we discussed above (Figure 2). This may cause additional oxidative damage due to involvement of ${\text{NADPH}}_{2}^{+}$ in enzymatic detoxification of reactive oxygen species. However, because an active mitochondrial FA synthase may use ${\text{NADH}}_{2}^{+}$ as a cofactor (Podack and Seubert, 1972; Seubert and Podack, 1973; Hinsch and Seubert, 1975; Whereat and Rabinowitz, 1975; Hinsch et al., 1976; Hiltunen et al., 2010; Smith et al., 2012) and H_2_ is readily transferred from ${\text{NADH}}_{2}^{+}$ to NADP^+^ by transhydrogenases (Bizouarn et al., 2000; Jackson et al., 2002), increased FA synthesis may not cause but rather protect against oxidative damage; **3**. Altered gene expression and protein modifications through increased protein acetylation. The ${\text{NADH}}_{2}^{+}$/NAD^+^ ratio modulates the activity of the NAD-dependent deacetylase sirtuin Sirt1 (Lin et al., 2004) that plays a central role in the regulation of thousands of metabolic enzymes and transcription factors in the cytosol and mitochondria (Hallows et al., 2006; Herranz and Serrano, 2010; Wang et al., 2010; Zhao et al., 2010; Hirschey et al., 2011); **4**. Decreased rates of oxidation metabolic reactions including the glycolytic pathway. Because over 700 oxidoreduction enzymes use NAD^+^ or NADP^+^ as cofactors (Sun et al., 2012), the reduced availability of oxidized NAD^+^ and NADP^+^ globally effects cellular biochemical processes; **5**. In addition, the altered reduction potential further reduces ATP production (Pettit et al., 1975), causing neuronal damage. Few mechanisms are involved in the reduction of ATP production including allosteric regulation and oxidized cofactor availability.

Because FA synthesis consumes H_2_ from two ${\text{NADPH}}_{2}^{+}$ per each acetyl-CoA incorporated into FA chain, H_2_ is readily transferred from ${\text{NADH}}_{2}^{+}$ to NADP^+^ by transhydrogenases (Bizouarn et al., 2000; Jackson et al., 2002), and an active mitochondrial FA synthase may use ${\text{NADH}}_{2}^{+}$ as a cofactor (Podack and Seubert, 1972; Seubert and Podack, 1973; Hinsch and Seubert, 1975; Whereat and Rabinowitz, 1975; Hinsch et al., 1976; Hiltunen et al., 2010; Smith et al., 2012), we hypothesized that increased FA synthesis under hypoxia has a role in maintaining cellular reduction potential. Importantly, under hypoxia each acetyl-CoA produced from glucose also produces two ${\text{NADH}}_{2}^{+}$. One is produced during anaerobic glycolysis, and another one during pyruvate oxidative decarboxylation. Thus, activation of FA synthesis will stoichiometrically use all ${\text{NADH}}_{2}^{+}$ produced in the anaerobic glycolysis and will prevent hydrogen accumulation in the form of lactic acid. In addition, it will support Glu oxidation also associated with NAD(P)${\text{H}}_{2}^{+}$ formation (McKenna, 2007). Using a loss of function approach through inhibition of FA synthesis at two different metabolic reactions at not-cytotoxic levels, we demonstrated a dramatic increase in ${\text{NADH}}_{2}^{+}$/NAD^+^ and ${\text{NADPH}}_{2}^{+}$/NADP^+^ ratios under hypoxia compared to vehicle-treated hypoxic neuronal cells (Figure 2).

FA synthesis inhibition also resulted in the increased lactic acid levels, and caused a significant toxicity under hypoxia but not normoxia (Figure 3). Because TOFA may alter a number of different pathways, it is difficult to provide a conclusive interpretation of the toxicity mechanism of fatty acid inhibition under hypoxia. However, because reductive potential was increased with both inhibitors under hypoxia, and increased reductive potential is the cause but not a direct result of apoptosis (Circu and Aw, 2010; Redza-Dutordoir and Averill-Bates, 2016), we speculate that decreased consumption of reduced cofactors in fatty acid synthesis pathway through inhibition may lead to cellular death under hypoxia.

Together, these data strongly indicate that FA synthesis is important for maintaining reduction potential and decreasing lactic acid, in order to support cell survival under hypoxia. These findings may help to identify a radically different approach to attenuate hypoxia related pathophysiology in the nervous system including stroke.

## Author contributions

SB: Conducted experiments, participated in designing the study and method development, writing and editing the manuscript, analyzed and interpreted the data. SG: Conducted MS experiments, participated in LC-MS method development and MS data analysis and interpretation. MG: Supervised and designed the study and method development, wrote and edited the manuscript, analyzed and interpreted the data.

## Funding

This publication was made possible by NIH Grant 1R01AG042819-04 (MG), NIH funded COBRE Mass Spec Core Facility Grant 1P30GM103329-04 (MG), and UND Office of the Vice President for Research funds (MG).

### Conflict of interest statement

The authors declare that the research was conducted in the absence of any commercial or financial relationships that could be construed as a potential conflict of interest.

## Abbreviations

FA: fatty acids
Gln: glutamine
Glu: glutamate
LDH: lactate dehydrogenase
MS: mass spectrometry
UPLC: ultra-pressure liquid chromatography.
